# Non-Secretory Multiple Myeloma: A New Observation and Review of the Literature

**DOI:** 10.7759/cureus.54479

**Published:** 2024-02-19

**Authors:** Zohra Ouzzif, Yassine Eddair, Wissal Laassara, Hicham El Maaroufi, El Mehdi Mahtat

**Affiliations:** 1 Biochemistry, Metabolic and Molecular Unit, Faculty of Medicine and Pharmacy, Mohammed V Military Training Hospital, Mohammed V University, Rabat, MAR; 2 Hematology and Immunohematology Laboratory, Faculty of Medicine and Pharmacy, Mohammed V Military Training Hospital, Mohammed V University, Rabat, MAR; 3 Department of Clinical Hematology, Faculty of Medicine and Pharmacy, Mohammed V Military Training Hospital, Mohammed V University, Rabat, MAR

**Keywords:** plasmacytosis, non-secretory, multiple myeloma, hematology, clinical biology

## Abstract

Non-secreting multiple myeloma is a rare variant of multiple myeloma that affects a relatively young population. It is characterized by the non-secretory nature of malignant plasma cells. The following case report describes the history of a 54-year-old patient with non-secretory myeloma revealed by mechanical and inflammatory low back pain. The bone and neurological involvement, the presence of diffuse osteolytic lesions and the increase in the serum kappa free light chains (FLC) level prompted a myelogram. This finds medullary invasion by dystrophic plasma cells. The absence of detection of complete or incomplete monoclonal immunoglobulin in the blood and urine, as well as the revelation of cytogenetic abnormalities of plasma cells, allows the diagnosis of non-secreting multiple myeloma to be made. This clinical case aims to describe the unusual presentation of this rare form of multiple myeloma.

## Introduction

Multiple myeloma (MM) is a hematologic malignancy characterized by abnormal proliferation of plasma cells and infiltration of the bone marrow. The disease is usually accompanied by hypercalcemia (C), renal failure (R), anemia (A) and bone lesions (B) known as CRAB criteria [1]. In 2-3% of MM patients, no identifiable monoclonal immunoglobulin can be detected in biological matrices [2]. This type of multiple myeloma, known as non-secretory multiple myeloma (NSMM), is defined by the presence of more than 10% clonal plasma cells in the bone marrow, or biopsy-proven plasmacytosis, evidence of organ damage related to the underlying plasma cell proliferation, particularly hypercalcemia, renal failure, anemia or bone lesions, and the absence of serum and urine monoclonal protein on electrophoresis and immunofixation [3,4]. Given its low frequency, few data are available on the diagnosis, clinical course and response to treatment of this type of MM. The present work aims to study the epidemiological, clinical and biological characteristics of NSMM through a case diagnosed and taken in charge at the Mohammed V Military Training Hospital.

## Case presentation

This is a 54-year-old patient with a pathological history of type 1 diabetes, arterial hypertension and benign prostatic hypertrophy under treatment. The history of the disease dates back to September 2022, with the progressive development of monoparesis of the right lower limb associated with mechanical low back pain. These became inflammatory and were complicated two months later by monoplegia of the same limb and urinary incontinence. The patient's general condition deteriorated and he lost 15 kilograms (kg) in five months. The patient was admitted to the rheumatology and then to the neurosurgery departments, before being transferred to the clinical hematology department for further management.

Clinically, the patient was conscious, apyretic and eupneic. Neurological examination revealed spinal cord compression syndrome. The results of the initial laboratory work-up are shown in Table 1.

**Table 1:** Initial biological workup

Biological parameter	Result	Reference values
White blood cells	7400 /µl	4000-10000
Hemoglobin	10.2 g/dl	13-17
Mean corpuscular volume	86 fl	82-98
Mean corpuscular hemoglobin concentration	34.4 g/dl	32-36
Platelets	346000/µl	150000-450000/µl
Reticulocytes	69000/µl	
Serum protein	67 g/l	64-83
Serum creatinine	10 mg/l	6-13
Glomerular filtration rate	86 ml/min/1.73 m^2^	
Serum Albumin	38 g/l	35-50
Calcemia	98 mg/l	80-105
Sodium	136 mmol/l	135-145
Potassium	5.30 mmol/l	3.70-5.30
Alkaline phosphatase	87 UI/L	40-150
Beta-2 microglobulin	2.76	0.97-2.64
Lactate dehydrogenase	169 UI/L	125-243 UI/L
Aspartate aminotransferase	12 UI/L	<35
Alanine aminotransferase	12 UI/L	<40

**Figure 1:**
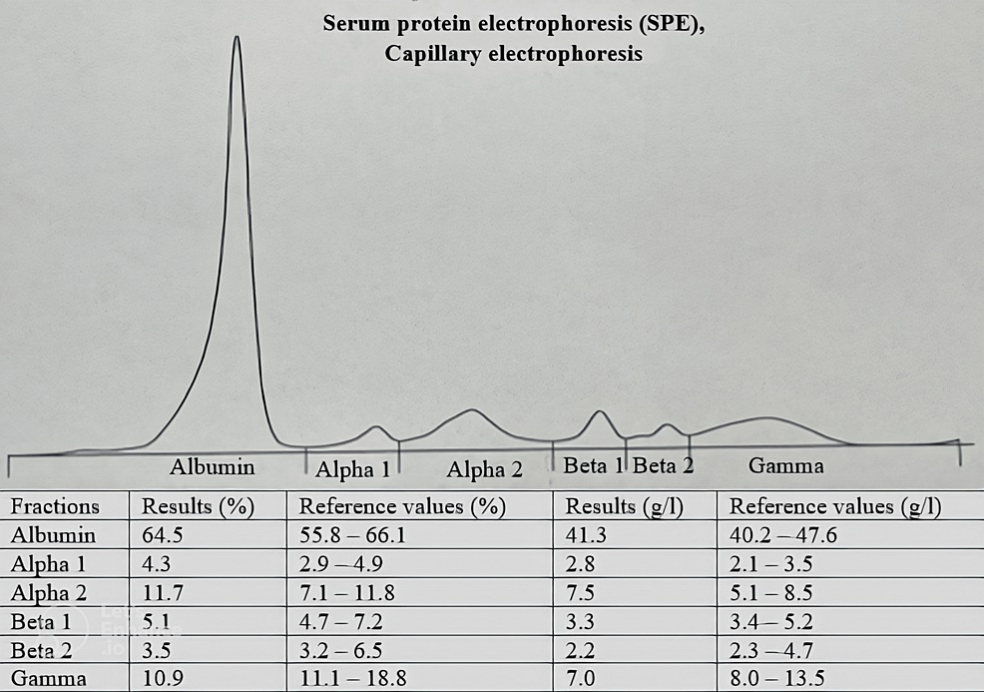
Serum protein electrophoresis. Total serum protein is 64 g/l The protidogram reveals normal levels of albumin, alpha1, alpha2, beta1 and beta2 globulin and a moderate hypogammaglobulinemia. No monoclonal immunoglobulin peaks were detected. Examination performed by capillary electrophoresis on the Sebia CAPILLARYS 2.

**Figure 2:**
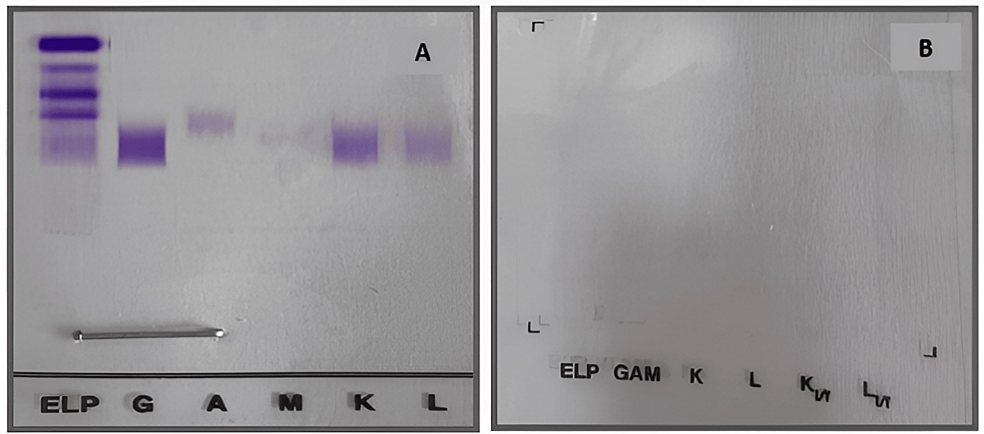
Immunofixation of serum (A) and urinary (B) proteins (Agarose gel, Sebia) No monoclonal component

The patient also underwent serum protein electrophoresis (SPE), which showed a moderate decrease in gamma globulins, and serum and urine immunofixation, which came back negative (Figures 1, 2).

**Figure 3:**
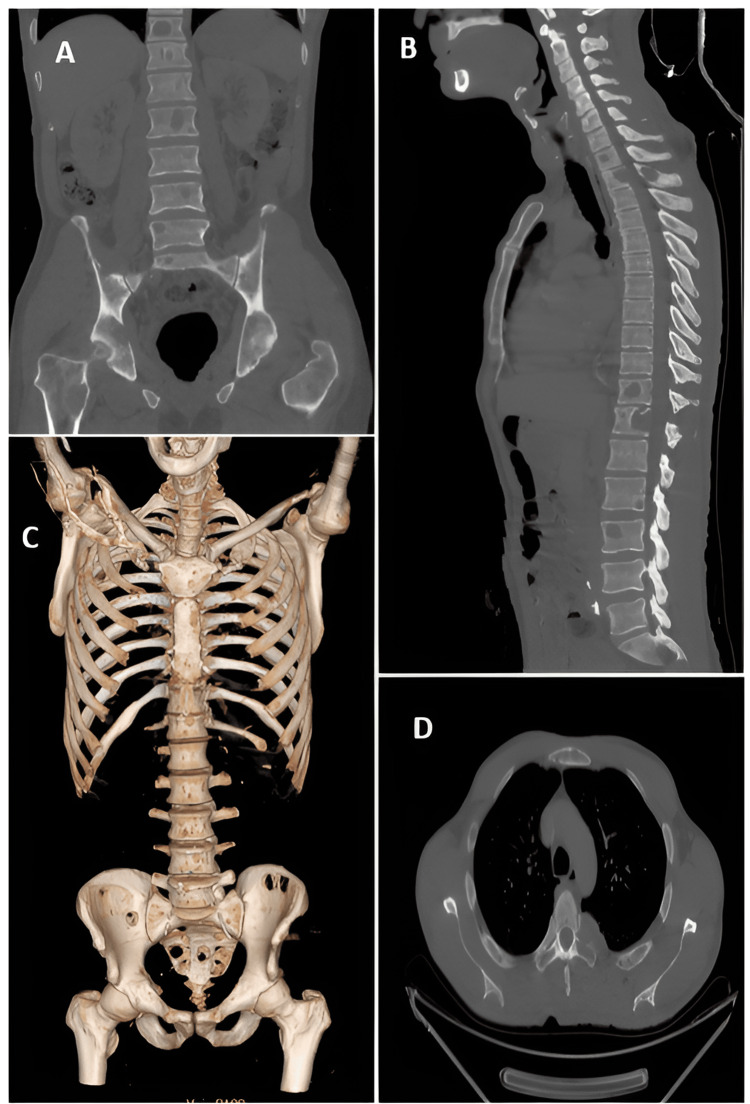
Thoracic-abdominopelvic computed tomography (CT) scan A&B- Sagittal and coronal bone window sections showing multiple lacunar bone lesions in the axial skeleton and pelvis. C- Thoracic-abdominal-pelvic CT scan with bone reconstruction showing the same lesions. D- Axial sections in the bone window: lytic lesions of the costal lacunae and transverse processes.

**Figure 4:**
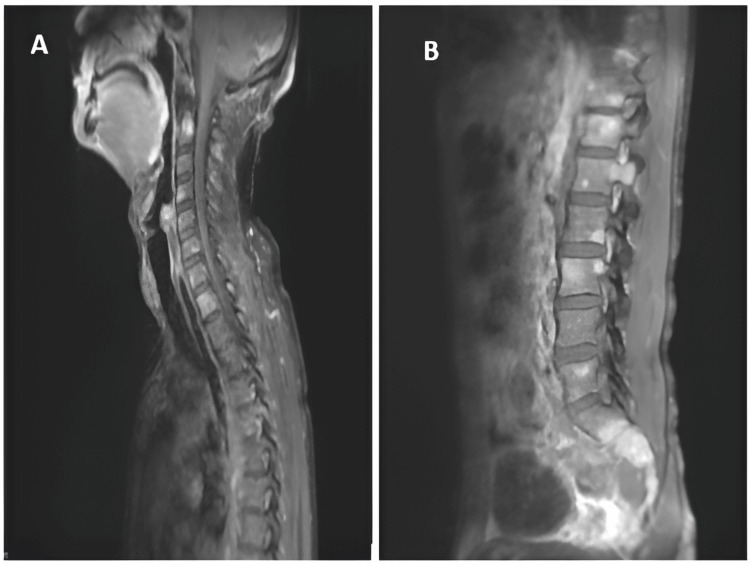
Spinal cord MRI A- Sagittal T1 acquisition with gadolinium injection showing multiple spinal lesions. B- T1 FS acquisition with gadolinium injection showing a lesion of the vertebral body from D12 and L1 to the posterior arch with epiduritis opposite.

On spinal cord magnetic resonance imaging (MRI), multiple secondary lesions extending to the posterior arches and soft tissues were revealed, in association with epiduritis on the D2, D5 and L1 levels. Complementary positron emission tomography with 2-deoxy-2-[fluorine-18] fluoro-D-glucose (18F-FDG PET scans) revealed multiple bony lytic foci scattered throughout the axial skeleton, with lytic masses in the posterior arch of left K5 and invasion of D5, L1 associated with extension to the left foramen and right sacral, non-fixing. There were no other suspicious pathological hypermetabolic foci on the rest of the structures explored from head to mid-thigh (Figures 3, 4).

In view of these clinico-biological and radiological findings, a serum free light chain (FLC) assay was carried out, showing an increase in serum kappa FLC and in the kappa/lambda (K/L) ratio, in the absence of renal damage (Table 2).

**Table 2:** Results of serum free light chains assay

Parameter	Patient results	Reference values
Kappa free light chains	50.94 mg/l	3.30-19.40
Lambda free light chains	12.93 mg/l	5.71-26.30
Ratio of free Kappa to free Lambda	3.94	0.26-1.65

**Figure 5:**
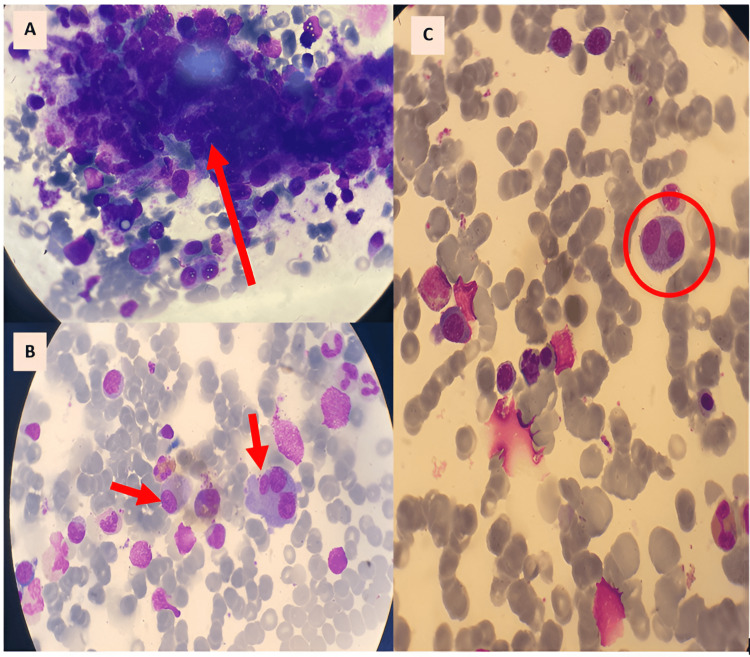
Major bone marrow invasion by atypical plasma cells A- Nest of plasma cells B&C- Plasma cells, medium to large in size, with basophilic cytoplasm, occasionally with irregular outlines, sometimes with two or three nuclei and loss of archoplasm (Olympus microscope).

A myelogram was performed, showing 64% invasion by highly dystrophic plasma cells, with the presence of plasma cell nests and rare megakaryocytes (Figure 5).

A cytogenetic and molecular study of bone marrow aspirates using Fluorescence In Situ Hybridization (FISH) revealed deletion of the Immunoglobulin Heavy Chain (IGH) locus at 11q32 in 68% of observed cluster of differentiation CD138+ plasma cells, absence of translocation t(4;14) in 100% of observed CD138+ plasma cells, absence of deletion of the Tumoral Protein (TP) 53 locus (17p13. 1) for 100% of observed CD138+ plasma cells, absence of amplification of the CKS1B locus at 1q21 and deletion of the CDKN2C locus at 1p32 for 100% of observed CD138+ plasma cells.

The International Staging System (ISS) score was 1 and the Revised ISS (R-ISS) score was also 1, indicating a low risk for the patient.

On the basis of these clinical and paraclinical findings, the diagnosis of non-secretory multiple myeloma was confirmed. Therapeutically, the patient underwent laminectomy of D2, VRD protocol (Velcade® (bortezomib) + Revlimid® (lenalidomide) + dexamethasone block), and 10 sessions of radiotherapy. The evolution was unfavorable and the patient died.

## Discussion

NSMM remains a rare condition and the literature presents mainly isolated observations of reported cases. Migkou et al. [2] reported in their work on a total of 852 patients, 100 (12%) cases identified as oligo-secretory/non-secretory of which 20 (2.3%) were true non-secretors. A Moroccan study of 443 MM cases identified five patients with NSMM (2.26%) [3].

NSMM cases were younger than those with MM [2]. The mean age was less than 65 years. Other studies [3,4] also concluded that NSMM patients were younger, with an average age of 57.80 years [3] and 56 years [4], respectively. In the present study, the patient was 53 years old, in line with cases reported in the literature. According to the International Myeloma Working Group (IMWG) criteria, MM is defined by the presence of clonal bone marrow plasmacytosis ≥ 10% (or histologically proven bone or extramedullary plasmacytoma) and at least one of the criteria attributed to plasma cell proliferation known as CRAB, acronym for hypercalcemia, renal failure, anemia, bone lesions (at least one osteolytic lesion present on skeletal X-rays, CT or PET scans).

Since 2014, three new criteria have been added to these, including a percentage of bone marrow plasma cells ≥ 60%, a serum free light chain ratio ≥ 100 (defined by the Binding Site test, with the affected free light chain needing to be ≥ 100 mg/L), or more than one focal lesion on MRI (at least 5 mm in size) [5].

Biological prognostic evaluation of MM cases includes serum albumin and β2-microglobulin, according to the International Staging System (ISS) prognostic score. In addition, lactate dehydrogenase (LDH) enzymatic activity and FISH cytogenetic analysis of sorted tumor plasma cells are performed. The latter two parameters are integrated into the Revised International Staging System (R-ISS) score [6]. As for NSMM, the IMWG has defined it as MM lacking monoclonal protein detected by serum and/or urine immunofixation, which may include light-chain MM with monoclonal FLCs detected only by serum FLC assay [7]. Since the plasma cell secretes an immunoglobulin (Ig) component, namely FLCs, cases of NSMM can be classified into at least two distinct groups with subcategories [1]:

The NSMM group (85% of cases) including: the oligo-secretory, FLC-restricted MM subgroup (light chains are detected using serum FLC assay in most cases, with serum monoclonal protein <1.0 g/dL, urinary monoclonal protein <200 mg/24 h and serum FLC values <100 mg/L), the true NSMM subgroup (plasma cells produce monoclonal protein, but are unable to secrete it into the extracellular space), the false NSMM subgroup (the immunofluorescence test shows the presence of monoclonal protein inside the plasma cell, but no extracellular monoclonal protein is found using standard laboratory tests).

Non-producing MM group (15% of cases): no Ig production by plasma cells.

The clinical presentation of NSMM remains a variable entity, with 10-40% of cases being asymptomatic and 50-70% presenting with bone pain or pathological fractures [8]. According to a British study [4], out of 10 cases, the diagnosis of NSMM was made after laminectomy-type surgery for neurosurgical clinical pictures.

In our case, the symptomatology was marked by low back pain, which led to his admission initially to rheumatology and then to neurosurgery for management of the spinal cord compression he presented. Biological and radiological examinations led to the diagnosis of NSMM.

Biologically, NSMM has its own particularities. CRAB criteria such as anemia, hypercalcemia and renal failure are rare or less marked [2]. The presence of lytic bone lesions was similar in patients with NSMM and those with secretory MM [9]. These data are in perfect agreement with the results of the present observation, which show renal function and blood calcium levels without abnormalities, whereas bone lesions were detected on radiological examination.

Serum protein electrophoresis reveals, in 80% of cases, hypogammaglobulinemia and the absence of a monoclonal peak [10], as in the case reported.

With the development of techniques for the detection of serum FLC by high-sensitivity enzyme-linked immunosorbent assay (ELISA), it has been demonstrated that most NSMM are probably oligo secretors, the monoclonal protein produced by MM plasma cells being restricted to FLC in the absence of heavy chains. Standard methods, such as SPE and serum IF, have difficulty detecting FLCs [11]. According to the literature, the serum FLC ratio is abnormal in 65% of cases at the time of diagnosis [2], as in the present observation.

NSMM patients also show less plasma cell infiltration of the bone marrow. Nevertheless, some studies have demonstrated bone marrow invasion by 52% of plasma cells with marked atypia. This is in line with the myelogram findings of the reported case [12].

With regard to the staging of multiple myeloma, NSMM patients are most often classified as stage ISS-1 or, when cytogenetic results are available, stage R-ISS-1 [9].

## Conclusions

Overall, NSMM represents a rare hemopathy whose biology remains partially understood. Serum FLC assays enable early diagnosis and evaluation of response to treatment. Monitoring of NSMM relies heavily on PET/CT scans and bone marrow samples. At present, therapy is similar to that for secretory MM, but NSMM-specific clinical trials would be extremely beneficial in improving patient management.
